# Indirect Measurement of Variables in a Heterogeneous Reaction for Biodiesel Production

**DOI:** 10.3390/mps7020027

**Published:** 2024-03-23

**Authors:** Ana Paloma González-García, Lourdes Díaz-Jiménez, Padmasree K. Padmadas, Salvador Carlos-Hernández

**Affiliations:** Sustentabilidad de los Recursos Naturales y Energía, Cinvestav Saltillo, Ramos Arizpe 259000, Coahuila, Mexico; paloma.gonzalez@cinvestav.edu.mx (A.P.G.-G.); lourdes.diaz@cinvestav.edu.mx (L.D.-J.); padmasree@cinvestav.edu.mx (P.K.P.)

**Keywords:** state estimation, heterogeneous catalyst, nonlinear model, batch reactor, soft sensor

## Abstract

This research focuses on the development of a state observer for performing indirect measurements of the main variables involved in the soybean oil transesterification reaction with a guishe biochar-based heterogeneous catalyst; the studied reaction takes place in a batch reactor. The mathematical model required for the observer design includes the triglycerides’ conversion rate, and the reaction temperature. Since these variables are represented by nonlinear differential equations, the model is linearized around an operation point; after that, the pole placement and linear quadratic regulator (LQR) methods are considered for calculating the observer gain vector *L*(x). Then, the estimation of the conversion rate and the reaction temperature provided by the observer are used to indirectly measure other variables such as esters, alcohol, and byproducts. The observer performance is evaluated with three error indexes considering initial condition variations up to 30%. With both methods, a fast convergence (less than 3 h in the worst case) of the observer is remarked.

## 1. Introduction

In the biodiesel industry, the monitoring of the transesterification reaction is required to avoid operation problems, ensure product quality, and detect failures [1]. The reactor is the central device of the production process; therefore, its supervision and control are highly recommended [2]. The variation in operating conditions in the reactor (temperature, reaction time, oil/alcohol molar ratio, and catalyst amount) affects the yield and quality of the product [3]. All of these parameters should be supervised throughout the reaction time to ensure that the biodiesel meets international standards.

Analytical procedures have been developed for evaluating biodiesel quality based on gas chromatography, liquid chromatography, nuclear magnetic resonance, and infrared spectroscopy [4,5]. These methods require samples to be taken and prepared for offline analysis, resulting in non-real-time transesterification monitoring. Furthermore, the industrial implementation of these techniques requires expensive equipment and qualified personnel to interpret the results, which is not suitable for online monitoring [4]. On the other hand, specific sensors have been developed to measure the concentration of biodiesel online. These sensors measure physical properties such as viscosity, refractive index, density, and speed of sound, which vary significantly for the compounds in the transesterification reaction mixture. These properties change during the transesterification reaction depending on the concentration of these species, which allows for monitoring the reaction [4]. However, as they are specialized sensors, their application is limited due to their high cost and difficult maintenance. In addition, they can introduce delays in the control loops [6].

**Figure 1:**
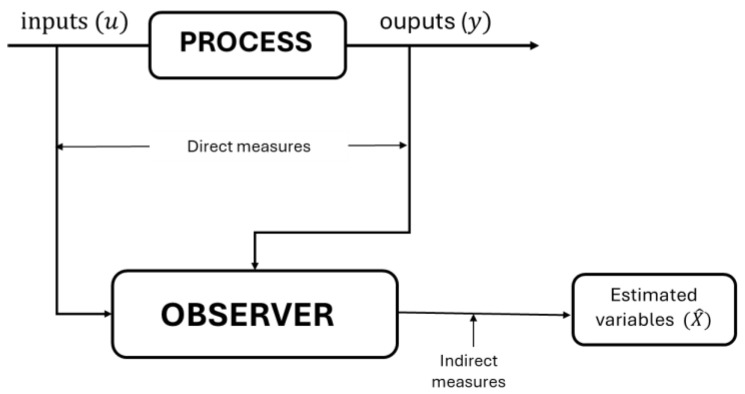
General scheme of state observer.

One of the alternatives to carry out supervision in real time and at affordable costs is to apply state estimation strategies, such as state observers. A state observer is a computational algorithm derived from a process mathematical model; this algorithm can reconstruct the evolution of state variables (whose measurement is technically difficult, expensive, or slow) from available variables (measured systematically). A general representation of a state observer is introduced in Figure 1.

The goal of the observer is to obtain an accelerated process dynamic, allowing us to predict (estimate) the behavior of variables; then, a mathematical model is required. Usually, the models considered are based on the general equation of the transesterification reaction [7] since it describes the different steps to transform triglycerides in biodiesel and crude glycerol, regardless of the reaction phase (homogeneous or heterogeneous transesterification); kinetics, conversion rate, reaction mechanism, and mass and energy balance are usually included in the models for variables’ estimation [5,8,9,10]. Once the models are formulated, it is necessary to adapt their structure according to the state space theory, since this is the basis for observer development. The main difference between homogeneous and heterogeneous transesterification is the reaction mechanism. The former is developed in a liquid phase and the reaction depends on the contact of triglycerides with the alcohol and the catalyst [11]; the second one takes place on solid/liquid phase and the reaction depends on the adsorption/desorption of reactants on the catalyst [12]. Thus, the corresponding models are different from each other due to the representation of said reaction mechanism. 

In recent years, artificial intelligence techniques (artificial neural networks, machine learning, and deep learning) are being considered to model the transesterification reaction [13,14,15,16]. These techniques try to process information as the human brain does; in this sense, no mathematical representation of phenomena is required, but rather computational algorithms. This allows us to model complex processes in a relatively easy way. In the case of heterogeneous catalysis, artificial intelligence models can be used to study the reactions [15], to predict behaviors, and to estimate variables [13,16]. The main issue of artificial intelligence is the need for a large quantity of data, which could be restrictive since several experiments should be performed which involves costs and time. 

Luenberger and Kalman introduced the basic concepts of state observers in the 1960s. Over time, research into state observer design became popular but challenging due to the high accuracy, low cost, and performance requirements. Nowadays, most current observers are modifications and extended versions of the classical Luenberger observer and the Kalman filter [17,18]. Regarding transesterification for biodiesel production, Table 1 summarizes the research related to the implementation of state observers.

The extended Kalman filter is used to obtain fast estimations based on probability theory; however, its implementation online can cause an excessive computational load because the method is complex [5,10]. On the other hand, observers based on artificial intelligence (Fuzzy and Takagi–Sugeno) are suitable for highly nonlinear systems with incomplete or unknown models. They also allow for formalizing and incorporating the empirical knowledge of the operators; however, their implementation online can be difficult and time-consuming, since they must first be adapted to the system [19,20]. An interval observer was implemented for a lab-scale reactor, achieving an estimation error of around 5%; this kind of observer provides variable estimations within an interval of values, assuming that initial conditions and uncertainties are unknown but bounded. The main issue is that the interval estimation error dynamic is required to be positive, which is not always accurate [9]. An evolutionary algorithm was used to minimize the estimation error in an algorithm devoted to estimating uncertain parameters; this algorithm was integrated into an automatic control strategy [21]. An unknown input observer [22] and a sliding-mode observer [23] for uncoupled multimodel representations of transesterification reactors were created; local observers were designed, and interpolation was used to recover the global dynamics. These kinds of observers allow us to consider uncertainties in the studied processes, but the design and tuning can become complex.

It is important to remark that the state observers have been used only in homogeneous reactions. No reports directly concerned with observers for heterogeneous transesterification have been found; only some works regarding the prediction of variables in heterogeneous reactions have been developed. For example, the prediction of the reaction yields was studied using an artificial neural network [15], and the use of machine learning techniques to predict the kinetic and thermodynamic behavior, as well as other aspects, of the heterogeneous reaction has been proposed [16]. Homogeneous transesterification is the most used reaction for biodiesel production at the industrial level [24,25,26]. However, operational and economic issues are induced since both the catalyst and the product are in the liquid phase. In homogeneous catalysis, the separation of the catalyst from the biodiesel represents additional costs and produces wastewater, which requires special treatment before disposal [26,27]. An alternative is the use of heterogeneous catalysts, which are in a solid state. Heterogeneous catalysts offer a series of advantages over homogeneous ones, such as being noncorrosive and safer to handle, and they do not wear out the reactors during processing. Furthermore, it is easy to separate them from the process by decanting or filtering and they can be reused; there is evidence that these catalysts can be reused in up to five cycles while maintaining their catalytic activity. Finally, heterogeneous catalysts can be made from low-value materials or from industrial waste, which positions them as sustainable materials [27,28,29,30]. However, the main disadvantage of heterogeneous transesterification is the decrease in the production rate, which is associated with mass transfer resistances, as the reaction takes place in a triphasic system (liquid–liquid–solid) [26,31]. To tackle these drawbacks, severe reaction conditions are usually applied [28]. In this sense, several studies have been performed to improve heterogeneous transesterification, but few works regarding state estimation have been reported. Therefore, the application of state observers to heterogeneous reactions represents an opportune area.

In this work, a state observer based on the Luenberger algorithm is developed for a heterogeneous transesterification reaction to estimate the dynamics of triglycerides, methanol, biodiesel, and glycerol concentration. Temperature measures are used as a key variable to perform the estimation. A mathematical model including mass and energy balance was previously obtained [32] and is used here to implement the observer. Two methods for the design of the observer are evaluated: pole placement and linear quadratic regulator (LQR). The observer is evaluated through numeric simulations, considering different operating conditions.

## 2. Materials and Methods

As said before, a state space representation of the transesterification reaction is required to formulate the estimation strategy. The state of a dynamic system is the smallest set of variables that allows us to determine the behavior of the system. The general representation in the state space of a nonlinear process is expressed by Equations (1) and (2):

(1) $$\dot{x}=f(x,u)$$

(2) y=h(x)

where *f* and *h* are smooth functions, *x* is the vector containing the state variables, *u* is the input vector, and *y* is the output vector. From this representation, the structure of a state observer is as follows:

(3) $$\dot{\hat{x}}=f\left(\hat{x},u\right)+L\left(\hat{x}\right)e=f\left(\hat{x},u\right)+L\left(\hat{x}\right)(y-\hat{y})$$

(4) $$\hat{y}=h(\hat{x})$$

where the circumflex accent denotes an estimated variable, and *L* is a vector called observer gain. Therefore, the observer design implies the selection of a gain *L*, which ensures a minimal estimation error. This error is defined as the difference between the real state and the estimated one, ideally $e=x-\hat{x}=0$. 

**Figure 2:**
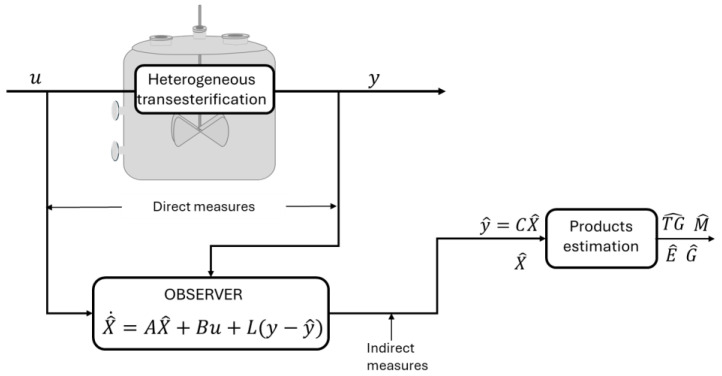
Schema of state observer for heterogeneous reaction for biodiesel production.

A schematic representation of the state observer proposed for the heterogenous transesterification reaction is presented in Figure 2. Reaction inputs (applied temperature) and outputs (reaction temperature) are represented by *u* and *y*, respectively; *X* (reaction rate and temperature), *A*, and *B* represent state variables, the state matrix, and the input vector, respectively, according to the state space representation. Finally, *L* is a vector that determines the dynamics of the observer.

### 2.1. Mathematical Model

The mathematical model required for the state observer was obtained from experimental data reported in a previous work [32], and each term is listed in Table 2. This model is based on the mass and energy balance of the transesterification reaction in a batch reactor [33,34]:

(5) $$\frac{dr}{dt}=-\left(\frac{1}{C_{0_{TG}}}\right)\cdot \left\{\frac{k_{1}\cdot \left(C_{M}^{3}-\frac{k_{2}\cdot C_{E}^{3}{\cdot C}_{G}}{C_{TG}}\right)}{{\left(1+k_{3\cdot }C_{E}\cdot \sqrt[{3}]{\frac{C_{G}}{C_{TG}}}+k_{4}\cdot C_{G}\right)}^{3}}\right\}$$

(6) $$\frac{dT}{dt}=\frac{-∆H_{R}}{\theta_{TG}C_{p_{TG}}+\theta_{M}C_{p_{M}}{+\theta }_{E}C_{p_{E}}+\theta_{G}C_{p_{G}}}\frac{dr}{dt}+\frac{UA\left(T_{A}-T\right)}{N_{0_{TG}}\left(\theta_{TG}C_{p_{TG}}+\theta_{M}C_{p_{M}}{+\theta }_{E}C_{p_{E}}+\theta_{G}C_{p_{G}}\right)}$$

In this model, the conversion rate (*r*) and the reaction temperature (*T*) are the state variables; the reaction temperature is taken as the output. To ease the handling of the model, it is rewritten as expressed by the following equations:

(7) $$\dot{x_{1}}=\frac{dr}{dt}=-\frac{x_{1}}{C_{0_{TG}}}$$

(8) $$\dot{x_{2}}=\frac{dT}{dt}=J\frac{dr}{dt}+Q=-J\frac{x_{1}}{C_{0_{TG}}}+Q$$

(9) y=T

The state equations are linearized to obtain a linear state space representation and to ease the implementation of the state observer. The Jacobian method is used to this end. Therefore, an equilibrium point is required: $\left\{x^{*},u^{*}\right\}:f\left(x^{*},u^{*}\right)=0$; the symbol * represents the value at the equilibrium. This equilibrium point implies that there is no variation in the state variables; it is obtained by setting Equations (7) and (8) as equal to zero and solving the resulting equation system. Once the equilibrium point is obtained, the matrices *A* and *C* for the state space representation are obtained as follows, with *f* representing Equations (7) and (8), and *h* being Equation (9):

(10) $$A=\frac{\partial f(x,u)}{\partial x}{│}_{x^{*},u^{*}}$$

(11) $$C=\frac{\partial h(x,u)}{\partial x}{│}_{x^{*},u^{*}}$$

### 2.2. Observer Formulation

The state observer structure is based on Equation (3), which is rewritten considering the notation for mass and energy balance of the heterogeneous transesterification reaction:

(12) $$\dot{{\hat{x}}_{1}}=-\frac{{\hat{x}}_{1}}{C_{0_{TG}}}+L_{1}\left(x_{2}-{\hat{x}}_{2}\right)$$

(13) $$\dot{{\hat{x}}_{2}}=-J\frac{{\hat{x}}_{1}}{C_{0TG}}+\hat{Q}+L_{2}\left(x_{2}-{\hat{x}}_{2}\right)$$

(14) $$\hat{y}={\hat{x}}_{2}=\hat{T}$$

where *L* = [*L*_1_ *L*_2_] is the observer gain. Considering the linearized model, the error $e=y-\hat{y}$, and $\hat{y}=C\hat{x}$, the state observer is also represented by Equation (15):

(15) $$\dot{\hat{x}}=A\hat{x}+Le=A\hat{x}+L\left(y-C\hat{x}\right)=\left(A-LC\right)\hat{x}+Ly$$

Since the reactor temperature is considered the output, the objective of the observer is to reach the condition $e=x_{2}-{\hat{x}}_{2}=0$ in finite time; then, the vector *L* is required to be selected to provide the observer dynamic to achieve a such condition. 

### 2.3. Computing of Vector L

The vector *L* was calculated using two methods: pole assignment and linear quadratic regulator (LQR). Both methods were applied using the software Matlab^TM^, R2020a.

The poles correspond to the eigenvalues of the state matrix of a system (matrix A). In practice, these values determine the dynamics of the process under study. Thus, the assignment of the poles of the observer (eigenvalues of [*A*−*LC*]) must be conducted to make it faster than the system. For this reason, it is recommended to make the poles of the observer 3 to 5 times larger (in absolute value) than the poles of the system to be observed. The choice of the desired poles determines the characteristics of the response obtained. In this sense, there can be an infinite set of vectors, but only a limited number of them meet the needs required for the system. It is advisable to test the response of the system to different values of poles chosen by the simulation. The command place() in Matlab was used for this goal.

The LQR method was also considered since it provides an optimal *L* vector. It is possible to calculate the optimal gain vector *K*, which allows the feedback u=−Kx to minimize the performance criterion $J\left(u\right)=\int_{0}^{\infty }x^{\prime }Qx+u\prime Ru$. Taking advantage of the duality property of the controllability–observability pair, it is possible to obtain the optimal vector *L* for the observer. The Matlab lqr() command was used, considering diagonal matrices *Q* and *R* with different weights. 

### 2.4. Observer Performance Assessment

The performance of the observer was evaluated through numerical simulations based on experimental data; the main criterion was the convergence of the estimated states toward the states calculated by the model. The initial conditions were varied between 5, 10, 20, and 30%. In each case, the observer performance was evaluated by the error indicators: *IAE* (integral of the absolute value of the error), *IAET* (integral of the absolute value of the error weighted with time), and *ISE* (integral of the square of the error), as defined in [35].

(16) $$IAE=\int_{0}^{\infty }\left|e(t)\right|dt$$

(17) $$IAET=\int_{0}^{\infty }t\left|e(t)\right|dt$$

(18) $$ISE=\int_{0}^{\infty }{e(t)}^{2}dt$$

where *e*(*t*) is the error defined as the difference between the estimated state and the real one.

### 2.5. Indirect Measures 

Once the reaction rate and temperature were estimated by the observer, the concentration of products was indirectly measured (estimated) by the following algebraic equations, which are obtained from the global reaction of transesterification [33]:

(19) $${\hat{C}}_{TG}=C_{0_{TG}}\cdot \left(1-\hat{r}\right)$$

(20) $${\hat{C}}_{M}=C_{0_{TG}}\cdot \left(\frac{{\hat{C}}_{M}}{{\hat{C}}_{TG}}-3\hat{r}\right)$$

(21) $${\hat{C}}_{E}=3\cdot \hat{r}\cdot C_{0_{TG}}$$

(22) $${\hat{C}}_{G}=\hat{r}\cdot C_{0_{TG}}$$

where ${\hat{C}}_{TG},\;{\hat{C}}_{M},\;{\hat{C}}_{E},$ and ${\hat{C}}_{G}$ are the estimated concentration in time (mol L^−1^) of triglycerides, methanol, esters, and glycerol, respectively; also, the subscript 0 stands for the respective value at the equilibrium point. In addition, $\hat{r}$ is the conversion rate (mol L^−1^ h^−1^) estimated by the observer.

## 3. Results

### 3.1. Observer Gain Calculation

By setting Equations (5) and (6) as equal to zero, the state $x=\left[r,T\right]=[0.98\;333]$ is obtained, and it is taken as the equilibrium point. Then, the linear state space representation of the transesterification model is described by Equations (23) and (24), where *A* and *C* are obtained by solving Equations (10) and (11).

(23) $$\dot{X}=AX=\left[\begin{array}{cc} -1.286 & 0.0084\\ -128.6693 & -20.9484 \end{array}\right]\left[\begin{array}{c} X_{1}\\ X_{2} \end{array}\right]$$

(24) $$Y=CX=\left[\begin{array}{cc} 0 & 1 \end{array}\right]\left[\begin{array}{c} X_{1}\\ X_{2} \end{array}\right]=X_{2}$$

where *X* represents the linearized states, $X=\left[\begin{array}{cc} X_{1} & X_{2} \end{array}\right]=\left[\begin{array}{cc} r_{linear} & T_{linear} \end{array}\right]$, and *Y* stands for the linearized output: *Y* = *T_linear_*.

To know the poles (*λ*) of the linear system, the eigenvalues of the state matrix are computed. For this case, the corresponding poles are as follows:

(25) $$\lambda =\left[\begin{array}{cc} \lambda_{1} & \lambda_{2} \end{array}\right]=\left[\begin{array}{cc} -1.3410 & -20.8934 \end{array}\right]$$

From these values, the vector *L* is calculated. As said before, the observer poles must be faster than the system poles to guarantee the reconstruction of the state variables. Since the poles are faster, they are more negative; in this work, “slow poles” are considered those that are very close to the poles of the system ($\lambda_{slow}≅\lambda_{system}$) and “fast poles” those that have a much lower value than the poles of the system ($\lambda_{fast}\ll \lambda_{system}$). Different pole assignments are considered to evaluate the behavior of the observer. First, the general recommendation (GR) is considered: the observer poles are three times faster than the system poles. Subsequently, a set of very slow poles [−1.5 −21] and one of very fast poles [−10.5 −147], as well as sets of combinations of them, are evaluated. The observer performance for each set of poles is evaluated by comparing the value of the IAET index (Table 3). It is observed that when using the set of poles *λ*_1_ = fast and *λ*_2_ = GR, the estimation error of both variables (conversion and temperature) is minimized. However, if the TG conversion is of greater interest, using the fast pole set is recommended.

**Figure 3:**
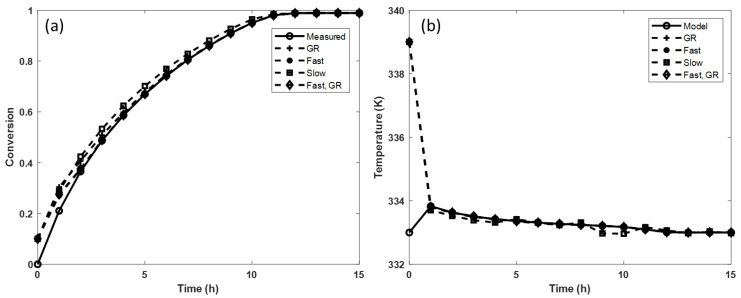
Observer performance considering different sets of poles (pole assignment method). (**a**) Triglycerides’ conversion estimation. (**b**) Reaction temperature. GR: poles with general recommendation, Fast: fast poles, Slow: slow poles, and Fast, GR: fast pole and general recommendation pole.

Figure 3 shows the result of the simulations when using different sets of poles. The estimation of the conversion rate provided by the observer is compared with the measurement (noted as measured) performed during the experiments. In addition, the estimation of temperature provided by the observer is compared with the one obtained by Equation 6 (noted as the model); since the mathematical model has been validated from experimental data [32], the calculated temperature represents that measured in the reactor. The selection of the observer poles induces differences in the estimation. Initial conditions of the observer different from those of the system have also been considered to evaluate the convergence of the estimation. It is observed that the set of poles [fast fast] and [fast RG] promotes rapid convergence of the state estimation (~3 h). In addition, the set of poles [RG RG] provides a good estimation of temperature, but not for the conversion; convergence is achieved after 7 h. Meanwhile, the response of the set of slow poles is the least favorable, both in the case of conversion and of the temperature of the reactor.

**Figure 4:**
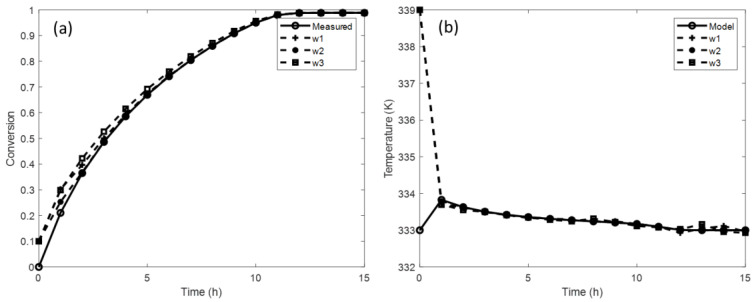
Observer performance considering different matrix weights (LQR method).

On the other hand, for the LQR method, it is not necessary to know the poles of the system; this advantage allows us to save a step in the observer design. As for the previous case, an iterative process is implemented. The weights of the matrices *Q* and *R* are taken as the starting point, where *w*_1_ = *w*_2_ = 1, respectively. Then, the weighting of *Q* is kept constant (*w*_1_ = 1), and the values of w_2_ are varied, and vice versa. Subsequently, the observer gain vector *L*($\hat{x}$) is calculated, and multiple tests are implemented to determine the performance of the observer. The results of these simulations are shown in Table 4 and Figure 4. It is remarked that as the weighting of *Q* increases, the error between the calculated states and the estimated states decreases.

Figure 4 shows the result of the simulations using different weights of the Q and R matrices. As in the case of pole assignment, the results provided by the observer are compared with the data from the experiments. The estimated rate conversion is compared with the measured one, and the estimated temperature is compared with that calculated by the model (Equation (6)). It is observed that when the weights *w*_1_ = 10 and *w*_2_ = 1 are used, the observer quickly converges both in the conversion (2 h) and in the temperature (1 h). Meanwhile, the weights *w*_1_ = *w*_2_ = 1 and *w*_1_ = 1 and *w*_2_ = 10 provide an acceptable response for the temperature estimation. 

### 3.2. Observer Performance Assessment

Observer performance is evaluated through simulations considering different initial conditions for both variables and by using the pole assignment and LQR methods. Since the conversion rate cannot be negative and its equilibrium value is zero, the variations in the initial condition for this variable are all positive; in addition, variations in the initial condition of temperature are both positive and negative in reference to the equilibrium value, which is 333 °F. The comparison between both methods is made based on the values of IAE, IAET, and ISE. The results of the simulations are presented in Table 5.

Regarding the conversion rate, even if the variations in T_0_ affect the magnitude of the indexes, it is possible to identify a direct relationship between the variation in the initial condition of the conversion rate and the value of each index: the larger the variation, the higher the index value. A similar effect can be observed on the estimation of temperature, as small variations (positive or negative) in T_0_ lead to small values of indexes, and large absolute variations produce large values of indexes. This implies that the observer achieves better estimations when the initial conditions are close to the operating point. However, for large variations in the initial conditions, the observer can perform the variable estimations with minimal errors, which is desirable. According to these values, both methods allow the observer to give good results when the initial conditions vary from 5 to 30%. However, since the computation of the observer gain with LQR is easier, it can be selected as the method for the observer design.

### 3.3. Estimation of Transesterification Variables

**Figure 5:**
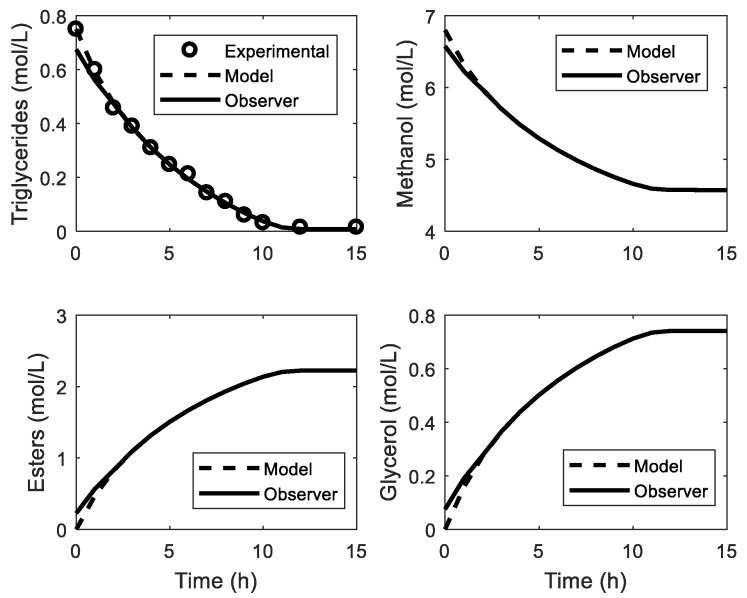
Estimation of the variables in the transesterification reaction by the Luenberger observer.

Considering the above, the behavior of the observer using LQR is presented in Figure 5. Values 10% higher than the real conditions of the process are taken as the initial conditions of the observer. The evolution of each of the components of the reaction mixture is presented. The observer rapidly converges, and it is possible to have estimates of each component after 2 h. On the other hand, the estimates of E and TG, which are typically the most important variables in the transesterification process, converge after 2 h and 1.5 h, respectively. In this way, it is possible to know its evolution in real time without the need for offline analysis.

## 4. Discussion

State observers have already been used as a strategy to estimate variables [5,9,19,20,21,22], parameters [21], and even the reaction heat [10] in the homogeneous transesterification of oils for biodiesel production. The estimated variables are used as soft sensors in supervision systems or as elements in control strategies [21]; the final objective is to enhance the performance of reactors for biodiesel production. All of the reported observers consider the measure of temperature. Temperature directly affects the phenomena involved in the reaction; therefore, in the mathematical models of transesterification, temperature appears as a key element related to the transformation of triglycerides in biodiesel. The temperature variation directly affects the conversion of triglycerides, which is greater when the reactor temperature is increased since a higher temperature causes a better interaction between triglycerides and methanol, promoting the reaction [36,37]. However, in this study, the temperature of the reactor remained constant (temperature increase was not applied), and a convergence between the observer and the model was expected to be achieved in less than two hours. Another measure reported for observers’ design is pH; the pH variations are directly related to the methyl esters concentration, which can indicate the reaction evolution. In a basic catalyzed reaction, the OH ions are available in the reaction mixture at the beginning because of their insolubility in oil. As the reaction elapses, the formation of FAME and glycerol produces a decrease in OH concentration in the reaction since OH ions are more soluble in the products. In a heterogeneous catalyzed reaction, the variation in pH obeys the acid nature of the triglycerides and biodiesel. The pH in biodiesel of high quality should be neutral, so it is possible to deduce biodiesel production from pH measures [20,37]. Finally, since the target products depend on the conversion rate, this measure is used to determine the relationships between products and parameters; for this reason, conversion rate has been used as a measure for a parameters observer [21]. It is worth mentioning that few results of the observation of heterogeneous reactions have been reported. From a mathematical viewpoint, modeling heterogeneous transesterification could be complex since the reaction is performed in a liquid–liquid–solid system; this induces challenges regarding the representation of phenomena taking place in the reactors [26,32,33,34]. Therefore, the results reported in this paper can guide future research on this topic.

The methodology of implementing an extended Luenberger observer to estimate the concentration of TG, M, E, and G throughout the transesterification reaction may be the easiest one and offers a balance between precision and complexity. In comparison with other methods such as the EKF [5,10,38], fuzzy methods [19,20], and multimodel methods [22,23], the computational load of Luenberger observers is lower since no recurrent calculations are required. The reported observers consider six [5,10,20] or four [19,21] differential equations; multiobservers consider two equations or set of models with two equations [9,22], but these are reduced models, and only two variables are estimated. The Luenberger observer presented in this paper considers only two differential equations instead of six or four as usual; the estimation of the concentrations of products is performed by algebraic equations. Thus, less computational load is required for the computation of the observer gain and for the variables’ estimation.

Regarding performance, the Luenberger observer achieves the estimation in around 1 h for the best case; the other cases allow for a convergence in less than 3 h. This behavior is similar to that obtained in other works [5,19,20] where the same variables are estimated. In addition, no undesirable oscillations are observed with this observer, as found in another report [19]. In addition, the performance index is comparable with that obtained for multiobservers [9,22,23]. It is important to remember that heterogeneous transesterification is considered in this work instead of the homogeneous one considered in the other reported works. 

In the studied transesterification reaction, rate conversion is the most restrictive variable since it requires offline analysis by chromatography. The reaction medium is sampled, the sample is prepared to be injected into a liquid chromatograph, and the result is analyzed to determine the conversion of triglycerides; this procedure takes around 30 min, and it must be performed each hour. The proposed observer allows us to know the conversion immediately once the temperature is known.

However, one of the main disadvantages of the Luenberger observer is that it requires in-depth knowledge of the characteristics of the process and the materials used. Likewise, Jacobians of the specific model describing the process are used to determine the gains of the Luenberger observer; this dependence could cause some divergence if there are changes in the model parameters.

Finally, from these results, it is suggested that the soybean oil transesterification process using a heterogeneous catalyst can be monitored by an extended Luenberger observer. TG measurements at different temperatures are sufficient to know the dynamics of heterogeneous transesterification and to estimate the concentration of triglycerides, methanol, biodiesel, and glycerol. However, it is necessary to complement the investigation with the validation of the behavior of the reactor temperature by means of real measurements. In this context, the results presented here are the basis for more complex supervision and control strategies. Future research could also integrate other variables, such as pH, that can be easily measured at a low cost and with robust sensors. It is feasible to consider other operating conditions (triglycerides’ source, catalyst, temperature, etc.) to expand the coverage of the observer. Also, it is possible to use this observer for fault detection procedures: if the observer produces anormal estimation of the variables, it can be associated with a failure in the reaction process. Finally, estimated variables could be used for automatic control schemes where all of the state variables are required online.

## 5. Conclusions

The extended Luenberger observer is adequate to estimate the evolution of the con-version of TG to biodiesel (E) and the temperature of the reactor, even when varying the initial conditions up to 30%. This offers an alternative to offline analyses such as gas chromatography analysis.

Temperature measurements can determine the dynamics of the heterogeneous trans-esterification process; however, in-depth knowledge of the characteristics of the process and the materials used is required, which can be considered a limitation or disadvantage of applying a state estimation strategy such as the extended Luenberger observer.

This study contributes to establishing a robust methodology for designing a state observer for people who do not have deep knowledge of mathematics or automatic control engineering.

## Figures and Tables

**Table 1:** Observers for transesterification for biodiesel production.

Observer	Measures	Estimated	System	Ref.
EKF	Temperature; pH	TG, DG, MG, G, and E	Homogeneous transesterification in CSTR	[5]
Fuzzy	Temperature	FFA, E, and water	Homogeneous esterification in CSTR	[19]
Fuzzy Reset	Temperature	FFA, E, and water	Homogeneous esterification in CSTR	[19]
Functional Fuzzy	Temperature; pH	TG, DG, MG, G, and E	Homogeneous transesterification in CSTR	[20]
Discrete Interval	Temperature	Fatty material; esters	Homogeneous transesterification in batch	[9]
EKF	Temperature	Reaction heat	Homogeneous transesterification	[10]
Evolutionary Algorithm	Temperature; conversion rate	Parameters	Batch homogeneous transesterification	[21]
Unknown Input Multimodel	Temperature	Fatty material; esters	Homogeneous transesterification in semi-batch	[22]
Sliding Mode	Temperature	Fatty material; esters	Homogeneous transesterification in batch	[23]
* Artificial Neural Network	Catalyst concentration	Esters yield	Heterogeneous transesterification	[15]
** Machine Learning	To be studied	Thermodynamic and kinetic data	Heterogeneous catalysis	[16]

TG: triglycerides, DG: diglycerides, MG: monoglycerides, G: glycerol, E: fatty acid methyl esters, and FFA: free fatty acids. * Presented as a prediction application. ** Presented as a perspective of machine learning in heterogeneous catalysis.

**Table 2:** Model parameters.

Name	Symbol	Value	Unit
Conversion rate	*r*	0 ^a^	mol·L^−1^·h^−1^
Triglyceride concentration	*C_TG_*	0.75 ^a^	mol·L^−1^
Ester concentration	*C_E_*	0 ^a^	mol·L^−1^
Methanol concentration	*C_M_*	6.8 ^a^	mL·L^−1^
Glycerol concentration	*C_G_*	0 ^a^	mL·L^−1^
Forward reaction constant	*k*_1_	1.72 × 10^5^	L^2^·mol^2^·h^−1^
Backward reaction constant	*k*_2_	2.34 × 10^−41^	-
Forward reaction constant	*k*_3_	2.46 × 10^−32^	L·mol^−1^
Backward reaction constant	*k*_4_	8.71 × 10^−19^	L·mol^−1^
Reaction temperature	*T*	333 ^a^	K
Reaction enthalpy	Δ*H_R_*	−260,718	J·mol^−1^
TG molar relationship	*θ_TG_*	0.0012	-
Methanol molar relationship	*θ_M_*	0.6061	-
Ester molar relationship	*θ_E_*	0.2945	-
Glycerol molar relationship	*θ_G_*	0.0982	-
TG specific heat	*C_pTG_*	3032	J·mol^−1^·K^−1^
Methanol specific heat	*C_pM_*	2785	J·mol^−1^·K^−1^
Ester specific heat	*C_pE_*	2234	J·mol^−1^·K^−1^
Glycerol specific heat	*C_pG_*	2556	J·mol^−1^·K^−1^
Heat transfer coefficient	*U*	511,200	J·h^−1^·m^−2^·K
Reactor area	*A*	0.0316	m^2^
Room temperature	*T_A_*	333	K
Limiting reactive initial mol	*N*_0*TG*_	0.285	mol

^a^ Corresponds to the initial condition.

**Table 3:** Observer performance considering the pole assignment method.

Observer Poles [λ_1_ λ_2_]	Observer Performance ^a^	
	TG Conversion	Temperature
[GR GR]	0.4251	0.8688
[slow slow]	1.4900	9.5167
[fast fast]	0.1034	0.2739
[slow GR]	1.4853	9.4845
[fast GR]	0.1138	0.2434
[GR slow]	0.4594	0.9528
[GR fast]	0.3809	1.3111
[fast slow]	0.1662	0.5908
[slow fast]	1.4688	7.761

^a^ According to the IAET index, and considering observer initial conditions as [*X*_1_ *X*_2_]_0_ = [0.1 339].

**Table 4:** Observer performance considering the LQR method.

Q and R Weight [*w*_1_ *w* _2_]	Observer Performance ^a^	
	TG Conversion	Temperature
[1 1000]	1.5861	12.3256
[1 100]	1.5193	9.5437
[1 10]	1.0216	2.2526
[1 1]	0.2755	0.8675
[1 0.1]	0.0697	0.2759
[0.1 1]	1.0216	2.2526
[10 1]	0.0638	0.1958

^a^ According to the IAET index.

**Table 5:** Observer performance considering different initial conditions and selected vector gain.

Observer Initial Conditions [*x*_1_ *x*_2_]_0_	Performance Index	TG Conversion		Temperature	
		Poles	LQR	Poles	LQR
[0.05 330]	IAE	0.0416	0.0485	1.5939	1.5809
	IAET	0.0440	0.05	0.7626	0.4934
	ISE	0.0013	0.0015	4.5011	4.5012
[0.05 336]	IAE	0.1141	0.0957	1.6597	1.6304
	IAET	0.2064	0.1328	1.1047	0.587
	ISE	0.00370	0.0033	4.5029	4.5035
[0.1 327]	IAE	0.0822	0.0946	3.1523	3.0991
	IAET	0.0881	0.0855	1.4727	0.4556
	ISE	0.0053	0.0058	18.0033	18.0017
[0.1 339]	IAE	0.2388	0.2029	3.1625	3.2331
	IAET	0.4251	0.2755	0.8688	0.8675
	ISE	0.0161	0.0147	18.0036	18.0137
[0.2 321]	IAE	0.1603	0.1812	6.1404	6.1589
	IAET	0.1388	0.1455	0.8482	0.6968
	ISE	0.0211	0.0229	72.0024	72.0054
[0.2 345]	IAE	0.5278	0.4635	6.3347	6.3696
	IAET	0.9814	0.6649	2.175	0.8015
	ISE	0.07530	0.0729	72.0131	72.0547
[0.3 315]	IAE	0.2388	0.2651	9.17	9.17
	IAET	0.2165	0.2071	1.34	0.51
	ISE	0.0474	0.0507	162	162
[0.3 351]	IAE	0.8732	0.7935	9.31	9.55
	IAET	1.6542	1.2307	1.36	1.14
	ISE	0.1983	0.2014	162	162

## Data Availability

Dataset available on request from the authors.
